# Implementing an Euregional databasis webtool for transborder surveillance of notifiable infectious diseases

**DOI:** 10.1186/2047-2994-4-S1-P230

**Published:** 2015-06-16

**Authors:** A Jurke, S Thole, M Lunemann, M Rohde, R Köck, K Soethoudt, A Friedrich, I Daniels-Haardt

**Affiliations:** 1 Infectiology and Hygiene, NRW Centre for Health, Münster, Germany; 2 R&D Division Health, Kuratorium OFFIS, Oldenburg, Germany; 3 Medical Microbiology, University Hospital Münster, Münster, Germany; 4 GGD Regio Twente, Twente, Netherlands; 5 Department of Medical Microbiology, Universitair Medisch Centrum Groningen, Groningen, Netherlands

## Introduction

There are surveillance data on notifiable infectious diseases at the European level. Despite the increasing cross-border mobility, there is no structure for the timely exchange of data on notifiable infectious diseases to date. Hence we need regional data for joint transborder action in infection control.

## Objectives

A webtool facilitating the transborder cooperation of public health stakeholders in infection control was developed in the Dutch German EurSafety Health-net project (http://www.eursafety.eu). Comparing the infectious disease reporting systems of The Netherlands and Germany we determined which routine data are available based on comparable case definitions on both sides of the border.

## Methods

Routine data on 11 notifiable infectious disease (hepatitis A, hepatitis B, hantavirus-infection, legionellosis, leptospirosis, listeriosis, measles, menigococcal disease, paratyphus, ornitosis, q-fever) can be imported i.e. weekly in the pilot euregional database tool. Based on historical data expected values are calculated. The deviation of current values from the expected ones (z-values) can be visualized, which may indicate clusters of infections.

## Results

For the first time infectious disease specialists of the public health services on both sides of the border can monitor the 11 notifiable infectious diseases of the border region up to the level of the local public health services in a joint system. In case of unusual cluster of notified infections they can quick contact each other to investigate the outbreak by a joint analytic study and close the uncommon source of infections.

## Conclusion

Surveillance of notifiable infectious diseases is developed in Europe at country level. Especially in border regions we timely need these data on regional level for transborder infection control. Useful surveillance of infectious diseases for transborder infection control can be based on routine notification data, standard software technology and should be easy to use and to maintain.

## Disclosure of interest

None declared.

